# A 326,000 fps 640 × 480 Resolution Continuous-Mode Ultra-High-Speed Global Shutter CMOS BSI Imager †

**DOI:** 10.3390/s25237372

**Published:** 2025-12-04

**Authors:** Jean-Luc Bacq, Mandar Thite, Roeland Vandebriel, Swaraj Bandhu Mahato, Philippe Coppejans, Jonathan Borremans, Linkun Wu, Kuba Rączkowski, Ismail Cevik, Vasyl Motsnyi, Luc Haspeslagh, Andreas Suess, Brandon Flon, Dan Jantzen, Phil Jantzen, Celso Cavaco, Annachiara Spagnolo

**Affiliations:** 1 IMEC, 3001 Heverlee, Belgium; 2 Apple Inc., Cupertino, CA 95014, USA; 3 Spectricity, 2800 Mechelen, Belgium; 4 Google Inc., Mountain View, CA 94043, USA; 5 Pharsighted, Parsippany, NJ 07054, USA

**Keywords:** CMOS image sensor, ultra-high-speed, continuous recording, backside illumination, global shutter

## Abstract

This paper describes an ultra-high-speed monolithic global shutter CMOS image sensor capable of continuous motion capture at 326,000 fps with a resolution of 640 × 480 pixels. The performance is enabled by a novel combination of pixel technology and circuit techniques. The highly sensitive BSI pixel with a 52 μm pitch employs a fully depleted substrate to facilitate rapid photocarrier transport. In-pixel voltage mode storage enables pipelined readout, while in-pixel analog CDS provides low noise with minimal impact on readout speed. The sensor achieves an equivalent row time of 6.4 ns through separate top and bottom readout together with multiple parallel ADCs per column. Independent row drivers on both the left and right sides ensure the global shutter accuracy needed for the minimum exposure time of 59 ns. The dynamic range is enhanced by on-chip reduction in FPN and by PTC-based data compression. The sensor delivers a throughput of 100 Gpix/sec, transferred off chip via 128 CML channels operating at 6.6 Gbps each. The device is fabricated using a 130 nm monolithic CIS process with BSI postprocessing and is in series production.

## 1. Introduction

Ultra-high-speed (UHS) global shutter image sensors are indispensable for realizing camera products capable of capturing very fast transient events. UHS imagers fall into two broad categories, namely, burst imagers and continuous recording imagers, each catering to different application niches.

**Figure 1:**
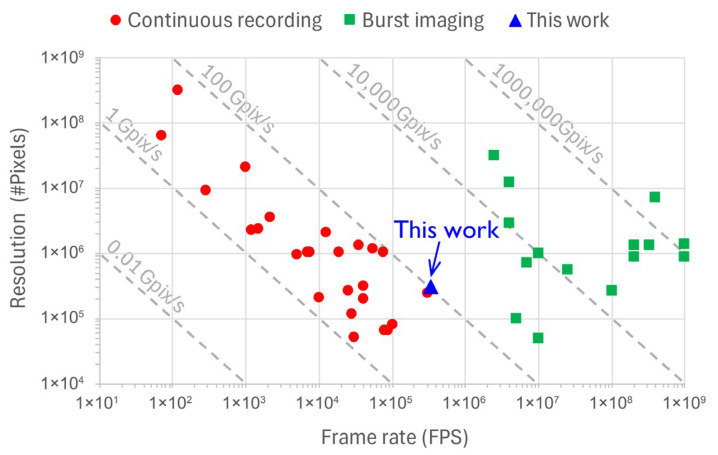
State-of-the-art high-speed imagers.

Burst imagers deploy in-pixel analog storage, thereby eliminating the need to read out the pixel array after every sensor exposure. This allows a short burst of frames at extremely high rates to be captured. Continuous recording imagers, on the other hand, have their pixel throughput limited by the sensor readout speed and output bandwidth. This introduces a fundamental trade-off between spatial resolution and temporal resolution offered by such imagers. A plot of spatial resolution versus frame rate (Figure 1) captures the performance evolution of high-speed imagers from the published literature, both commercial and scientific. It shows significant advancements in the performance of CMOS imagers where continuous recording capability is being offered at frame rates that easily exceed tens of thousands of FPS. As per the plot, the boundary between continuous and burst imagers occurs at the pixel rate of 100 Gpix/s and the sensor reported in this paper finds itself at this very forefront of continuous high-speed imaging. With a novel combination of advanced pixel technology and circuit techniques, this sensor offers a throughput and sensitivity performance that exceeds the state of the art in continuous recording imagers.

## 2. Imager Architecture

### 2.1. Pixel Array

**Figure 2:**
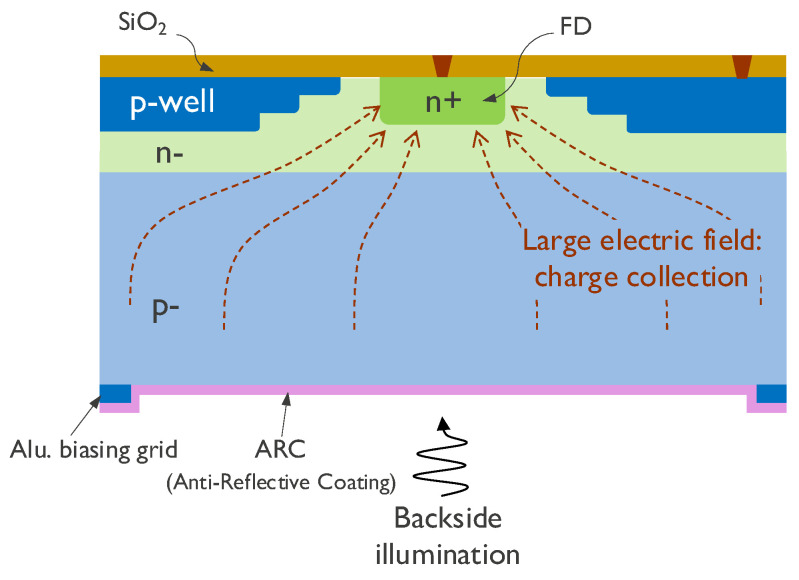
Pixel diode design.

The sensor uses pixel technology and in-pixel circuit techniques specifically developed for high-speed operation [1,2]. The pixel diode cross-section is shown in Figure 2. The pixel is backside-illuminated (BSI) with anti-reflective coating (ARC) and consists of a fully depleted substrate. While BSI and ARC lead to a high fill factor and quantum efficiency, the large electric field in the substrate allows for the fast collection of photocarriers while minimizing pixel-to-pixel crosstalk. This results in a highly sensitive pixel. To boost the frame rate, the pixel incorporates two circuit techniques, namely, in-pixel storage for pipelined readout and in-pixel CDS for kT/C noise suppression with minimal speed loss.

**Figure 3:**
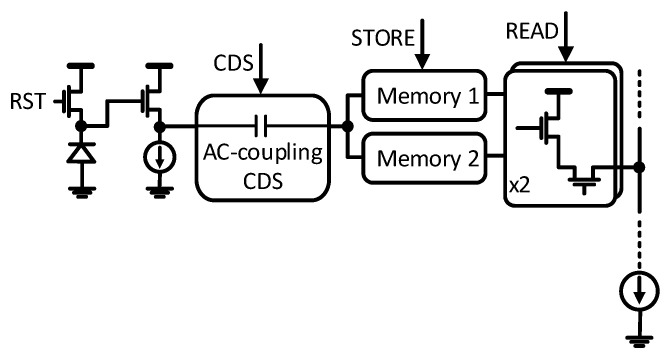
Pixel schematic.

**Figure 4:**
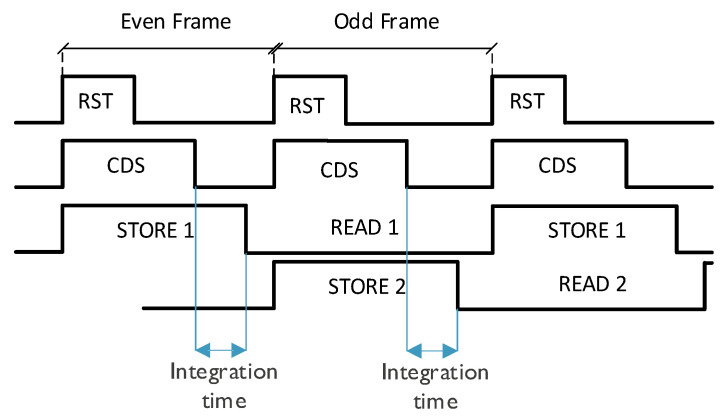
Pixel timing diagram.

Realizing an electronic global shutter requires in-pixel storage to hold the image frame while it is read out. With extra storage, the pixel can hold multiple frames, thus allowing the pipelining of integration and readout to achieve a higher throughput. With the same goal, this sensor uses an implementation of dual in-pixel voltage mode storage [3], as depicted in the pixel circuit diagram in Figure 3. It allows the integration of the new frame and readout of the previous frame to happen in parallel, as shown in the timing diagram in Figure 4.

The pixel array is read out in a rolling fashion. With the full ROI frame rate of 326,000 fps and with 480 rows per frame, the effective row readout time is 6.4 ns. As described in Section 2.2, the sensor deploys 16 ADCs/column to achieve the target readout time. This means one full frame readout is composed of 30 A/D conversion cycles, with a single A/D conversion period being 102 ns (corresponding to a sample rate of 10 MSPS per ADC).

Low temporal noise is critical for ensuring sufficient signal sensitivity especially in imagers with exposure times as short as 59 ns (this work). CMOS imagers commonly rely on digital CDS for pixel kT/C noise suppression which requires two readouts per pixel. The in-pixel CDS [4,5] allows this sensor to perform analog CDS within a single readout, thus relaxing the noise–speed trade-off. As shown in Figure 3, the CDS is based on AC coupling. The sampled kT/C_FD_ noise is treated as a DC component that is blocked by the CDS stage. As a result, only the photogenerated signal swing couples to the output.

**Figure 5:**
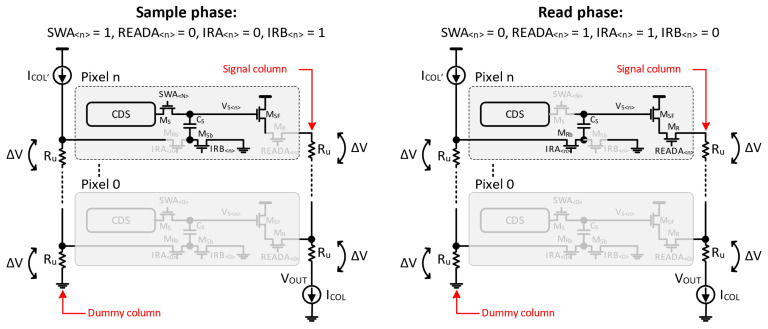
Concept of vertical gradient compensation.

With 480 rows and a 52 μm pixel pitch, the pixel column is as high as 2.5 cm. To achieve sufficiently fast column settling over such a large physical dimension, a high column bias current value of 50 μA is used. With the column metal line parasitic resistance on the order of 1 kOhm, the bias current creates a top-to-bottom IR drop of about 50 mV, which is 10% of the signal dynamic range. To avoid losing part of the dynamic range to vertical gradient, the sensor incorporates a mechanism to compensate the column IR drop [6]. In this scheme, a dedicated dummy column metal line with the exact same dimensions as the signal column line is added to every pixel column. As depicted in Figure 5, a voltage reference equal to the column IR drop is provided to each pixel via the dedicated dummy column having identical routing and bias current as the signal column. The bottom plate of the in-pixel sampling capacitor is connected to the pixel ground during sampling and it is switched to the dummy column during readout. This effectively removes column IR drop from the signal at the end of the column before A/D conversion.

### 2.2. Sensor Architecture

**Figure 6:**
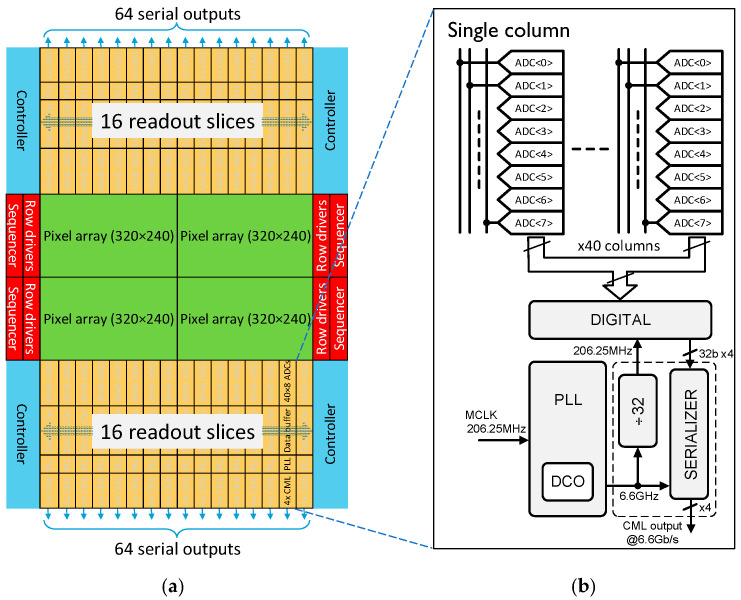
(**a**) Top-level architecture; (**b**) single readout slice.

The challenge of achieving very high frame rates with the large pixel array dimensions (3.3 cm × 2.5 cm in this work) is addressed by extensive parallelization of the column readout [7,8]. As shown in Figure 6, the sensor uses separate top and bottom readouts with eight ADCs per column at a 52 μm pitch. Both the top and bottom sets of the ADCs simultaneously convert 8-row blocks per column, reducing the row readout time by 16 times. The top ADCs handle the upper half of the array, while the bottom ADCs process the lower half. With 640 columns and 16 ADCs per column, the sensor features a total of 10,240 on-chip ADCs.

To achieve global shutter accuracy for minimum exposure time, separate row drivers are added to the left and to the right of the array [7,8], controlling the respective halves. This creates four independent pixel array quadrants, each with its own row control and readout circuitry (Figure 6a). Synchronization of the quadrants is handled at the application level through the careful distribution of trigger and clock signals on the camera PCB.

The readout is organized in slices mapping to 40 columns. Figure 6b shows a block diagram of one slice which consists of 320 A/D converters followed by digital logic to perform data compression and encoding for serial transmission via 4 high-speed CML channels.

**Figure 7:**
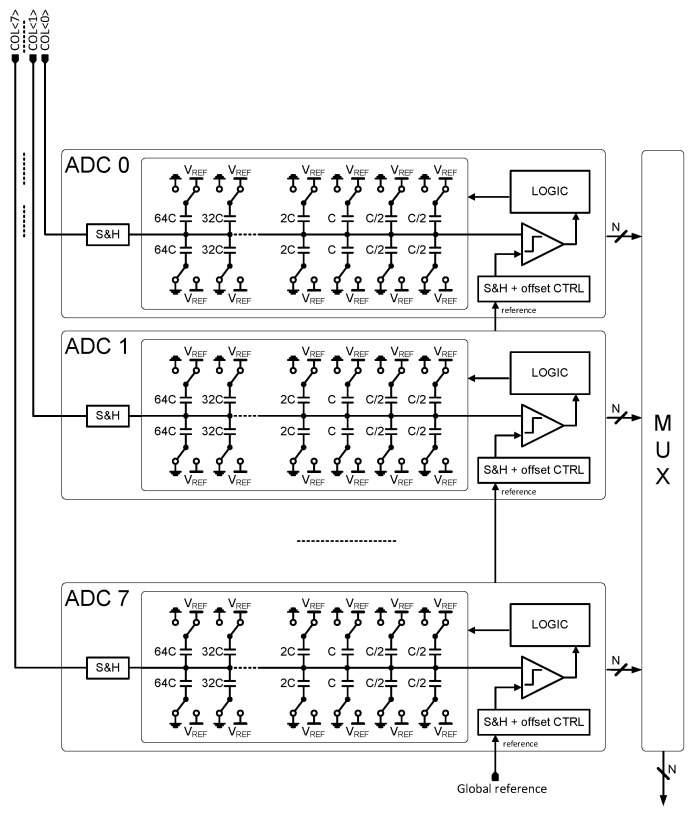
ADC block diagram.

The ADC is a low-power asynchronous SAR that achieves 9-bit resolution at 10 MSPS throughput, consuming 500 µW. A block diagram is shown in Figure 7. It uses a charge redistribution capacitive DAC with a custom MOMCAP layout. An external voltage is provided to each ADC as a comparator reference. It is sampled locally, similar to the column input, and it can be calibrated individually in each ADC to compensate for ADC offset mismatch.

The four serializers require a robust, low-jitter clock signal at 6.6 GHz which is generated by a PLL. To ensure the quality of the clock signal at an RF frequency, it is crucial to minimize the signal fan-out and routing distance. This is achieved by including a dedicated PLL in each slice, resulting in 32 PLLs across the chip overall.

**Figure 8:**
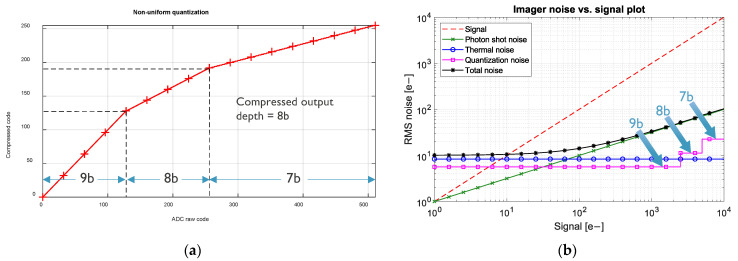
Non-uniform quantization: (**a**) ADC data compression; (**b**) modeled imager noise.

To reduce the data transfer rate, the 9-bit outputs of the ADCs are compressed into 8-bit words exploiting the shot noise-limited SNR of the imagers at high signal levels [9]. Figure 8a shows the transfer characteristics of the digital encoder. The signal range is split into 3 regions. Starting at 9-bits in the lowest signal range, the number of bits is reduced by 1 for each of the subsequent higher signal ranges. This causes the RMS quantization noise to increase by 2× and 4× in the two high signal ranges. However, as seen in Figure 8b, the quantization noise remains well below photon shot noise over the entire signal range, which allows for practically lossless data compression. Output data of multiple ADCs are interleaved in continuous serial bit streams which are converted to 64B/66B Aurora protocol frames and transmitted over 6.6 Gbit/s CML outputs. The sensor packs 128 of these CML outputs in total.

**Figure 9:**
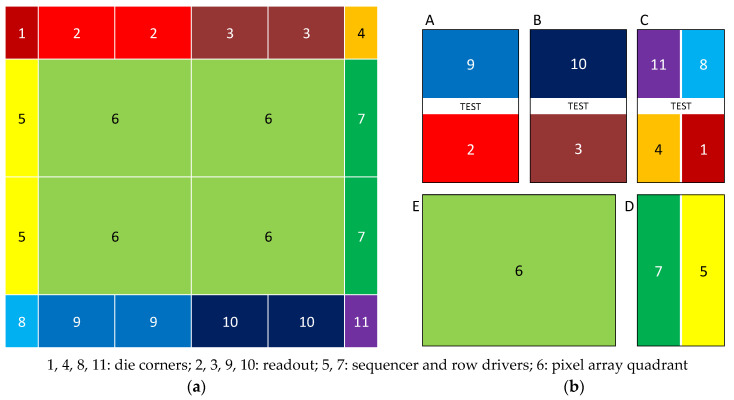
Fabrication by stitching: (**a**) die composition; (**b**) reticle composition.

As the overall chip size is larger than the reticle size, the chip is fabricated by stitching together smaller blocks that fit within the reticle. Figure 9a shows the sensor die split into various functional blocks (1 through 11), whereas Figure 9b shows these functional blocks arranged in 5 separate stitching blocks (A through E) of the reticle, each of which is translated and projected onto the wafer.

## 3. Measurement Results

**Figure 10:**
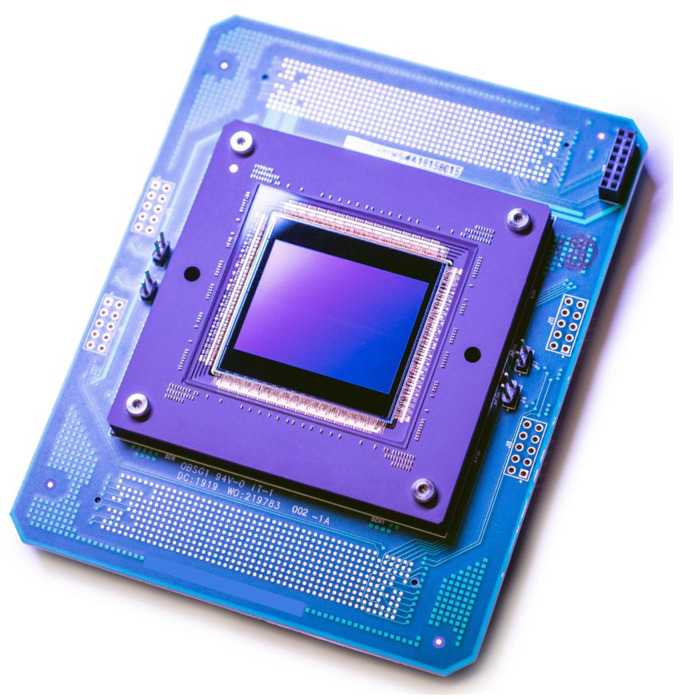
Packaged BSI sensor.

The sensor described here has been silicon verified and is in series production. Figure 10 shows the packaged sensor mounted on a PCB that is designed to be used in the camera as well as on the testbench.

**Figure 11:**
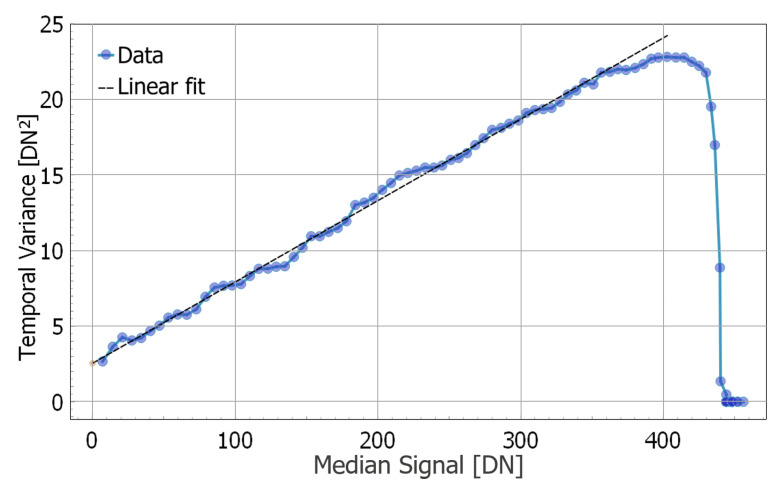
Photon Transfer Curve (PTC) measurement.

The basic characteristics of the imaging sensor are extracted using the well-known method of the Photon Transfer Curve (PTC). An example of such characterization is shown in Figure 11. Here, sets of 100 frames were captured at different integration times under a fixed illumination level. The temporal variance of pixel values is compared to the median pixel value. The slope of linear fit to the data corresponds to the Conversion Gain (CG) of 0.054 DN/e- and offset to Read Noise (RN) of 1.6 DN, or 29.5 e- RMS. A Full Well (FW) capacity of 402 DN or ~7500 e- is inferred from the position of the PTC maximum. Also, as one can see, the PTC shows some ripples around the linear fit. These ripples are thought to be a combined result of random fluctuations in the spatial mean values of successive frames and higher temporal variance around the crossover points of the three data compression regions (see Figure 8). The exact causes of this effect are not yet completely understood.

**Figure 12:**
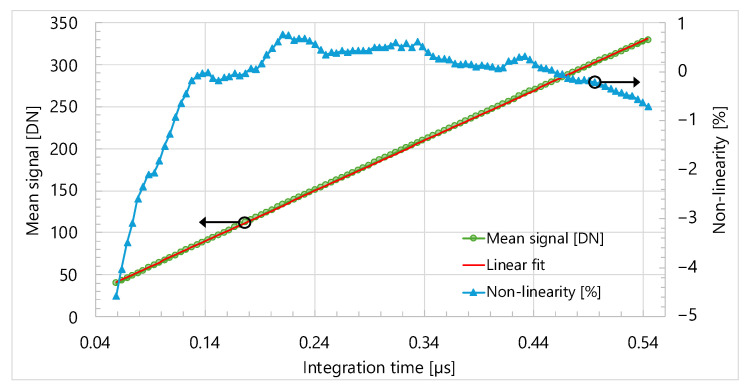
Sensor linearity vs. integration time.

Similarly to PTC, sensor linearity is also assessed by capturing image frames under constant illumination while varying the integration time. Figure 12 shows a plot of mean signal level per frame versus integration time based on this data. Non-linearity is computed as the percentage deviation of the mean signal versus the integration time curve from its linear fit. The results indicate that absolute non-linearity remains below 5% for integration times as short as 59 ns.

**Figure 13:**
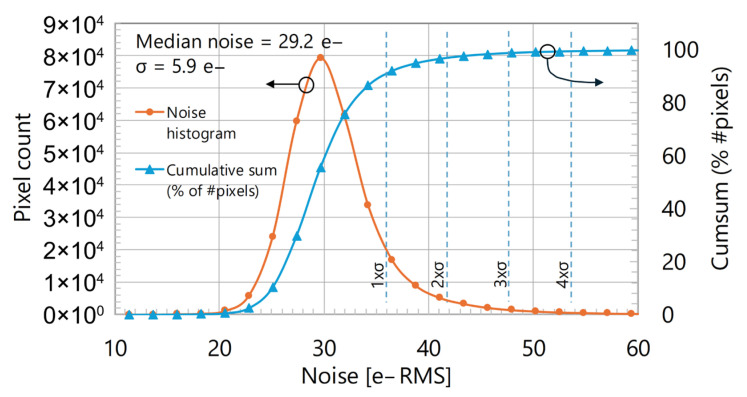
Temporal readout noise histogram.

Figure 13 displays a histogram of temporal readout noise. The plot indicates that 99.2% of the pixels have a noise level within four sigma. Compared with the original pixel performance reported in [1], the sensor described here exhibits a higher Read Noise. This difference is due to the fact that [1] only addresses the pixel noise (~7 e^−^_rms_), whereas this work reports the total noise (~29 e^−^_rms_) consisting of both the pixel noise and the ADC noise. The ADC is the dominant noise source with a target noise spec of 14.5 e^−^_rms_, which translates to an overall sensor noise spec of ~16 e^−^_rms_. Although, on actual silicon, the overall noise level is measured to be ~29 e^−^_rms_, which is higher than the target spec of 16 e^−^_rms_. This is attributed to a higher noise contribution from the ADCs mainly caused by the power supply crosstalk.

**Figure 14:**
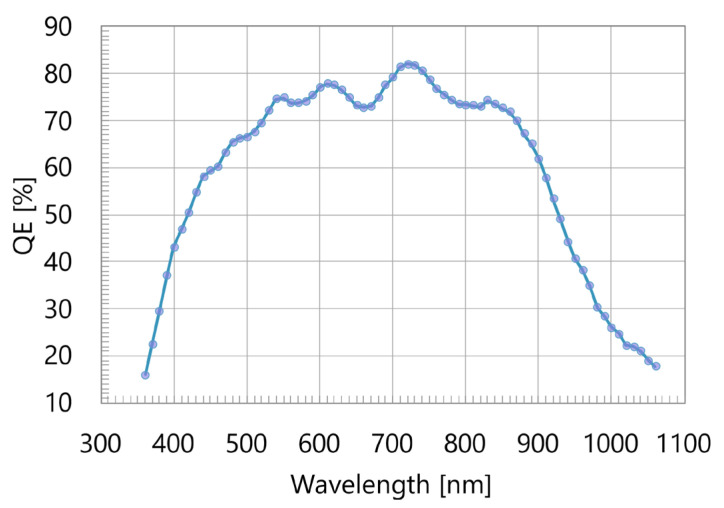
Quantum efficiency vs. wavelength.

For the spectral characterization, the imager was illuminated by a uniform monochromatic light source of known intensity. For this purpose, a narrow spectral line (FWHM ~10 nm) from a broadband light source was selected using a monochromator and was coupled into an integrating sphere. The sphere provided uniform illumination across the sensor plane. For each spectral point, a set of 100 images was recorded for each value of a set of different integration times. A linear fit to the median of pixel values vs. integration time was used to extract the photoresponse value in the units of DN/s. A comparison of this photoresponse to the known photon flux allows for the calculation of Quantum Efficiency (QE) for each spectral point. An example of such a spectral characteristic is shown in Figure 14. Thanks to BSI, the sensor evidently achieves a high QE over a broad spectral range. Some oscillations of the QE values are thought to be due to the Fabry–Perot etaloning effect in the Si absorber layer.

**Figure 15:**
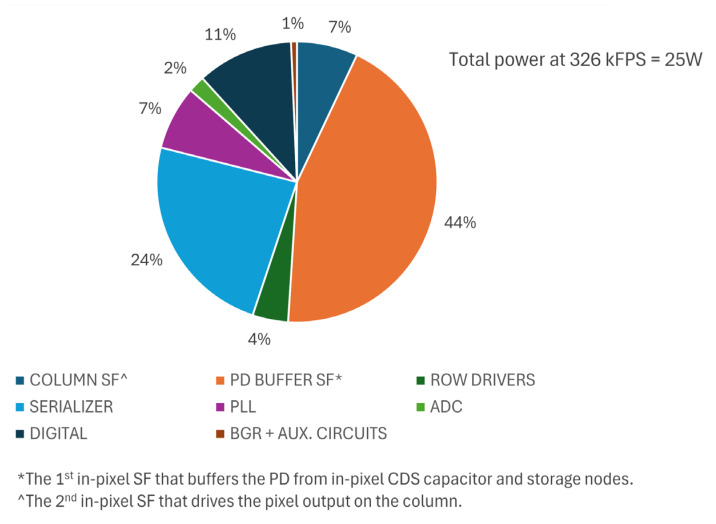
Power consumption distribution.

The pie chart in Figure 15 shows the distribution of overall power consumption across various functional blocks of the sensor chip. The top consumers are the pixel source followers (at 44%) and serializers (at 24%). A substantial power budget is spent on the pixel internal source follower to meet the stringent pixel timing requirements, namely, a min. pixel reset time of 300 ns and a min. integration time of 59 ns. A total of 128 CML serial data channels constantly clocked at 6.6 GHz makes serializers the second biggest consumer.

The key performance specifications of the sensor are listed in Table 1. With an EMVA 1288 absolute sensitivity threshold of 0.011 e-/µm^2^ and a frame rate of 326,000 fps, the sensor advances the state of the art [7,10] with respect to these metrics.

**Figure 16:**
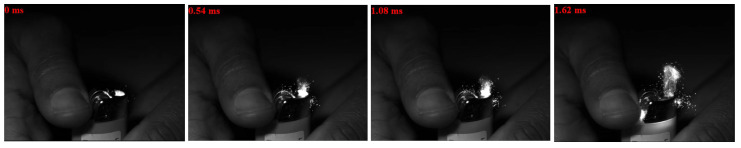
Four non-successive 640 × 480 frames from a 240,000 fps video of lighter ignition at 2 µs exposure.

**Figure 17:**
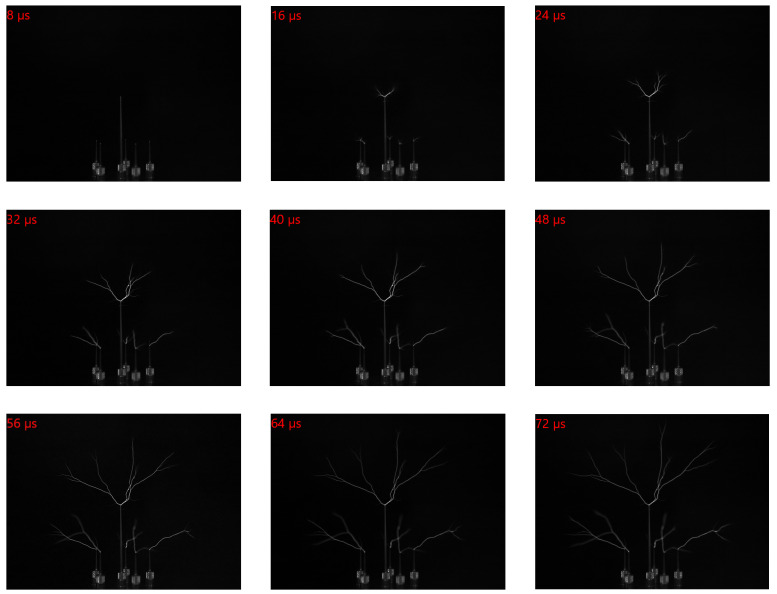
Non-consecutive 640 × 480 frames from a 250,000 fps video at 1.69 µs exposure show electric discharge arcs produced by a Tesla coil at several tens of kilovolts.

Two examples of ultra-fast transient events captured by the sensor in a high-speed camera are presented next. Figure 16 shows four non-consecutive frames from a 240,000 fps video depicting an igniting lighter. Similarly, a selection of nine non-consecutive frames that capture arcing due to electric discharge around a Tesla coil at 250,000 fps is shown in Figure 17. These examples demonstrate the imager’s performance.

## 4. Conclusions

A 640 × 480-pixel monolithic ultra-high-speed and high-sensitivity continuous recording global shutter image sensor fabricated using a 130 nm CMOS BSI process has been demonstrated. With respect to sensitivity and throughput, the sensor’s performance surpasses existing continuous recording high-speed imagers, placing it among the fastest in this class.

The sensor achieves its high-speed operation through dedicated pixel technology together with both in-pixel and off-pixel circuit techniques. Low input referred noise, combined with the fully depleted substrate and BSI postprocessing, allows the sensor to attain high signal sensitivity.

The sensor thus reported is the beating heart of a range of high-speed camera systems currently available, enabling a host of scientific high-speed imaging applications.

## 5. Patents

The technique of column IR drop compensation (outlined in Figure 5) to reduce the column FPN resulted in United States Patent US 10,778,926 B2 [6].

## Figures and Tables

**Table 1:** Performance comparison.

Parameter	This Work [11]	[7]	[10]
Technology	130 nm, 5 metal CMOS, BSI	110 nm, 6 metal CMOS, BSI	CMOS, BSI
Binning	Not available	2 × 2	2 × 2
Resolution	640 × 480	1280 × 832	Binned 640 × 384
Pixel pitch	52 µm	18.54 µm	Binned 37 µm
Shutter type	Global	Global	Global
FWC	7.5 ke-	N/A	33 ke-
Conversion Gain	0.054 DN/e-	N/A	N/A
QE @ 532 nm	72.7%	N/A	72.0%
Max. FPS (max. ROI)	326,000	80,000	308,820
Equivalent row time	6.4 ns	15.0 ns (no binning)	8.4 ns
Readout noise	29.5 e- RMS	N/A	70.7 e- RMS
μ_e.min.area_ ^‡^ (EMVA 1288)	0.011 e-/μm^2^	N/A	0.056 e-/μm^2 †^
Min. integration time	59 ns	Binned 95 ns	95 ns/38 ns
Dynamic range	48 dB	52 dB	53.4 dB
Bit depth	9 bits	10/11/12 bits depending on row time	12 bits
Output channels	128 channels @ 6.6 Gbps	160 channels @ 6.25 Gbps	N/A
Image lag	1 DN (9-bit)	N/A	N/A
Non-linearity	<5%	<1.75%	1.29% ^†^
Dark current @ room temperature	3.41 nA/cm^2^	N/A	2.3 nA/cm^2 †^
Power consumption	25 W	<40 W	N/A

^†^ Extracted from EMVA 1288 report for binned mode. ^‡^ Absolute sensitivity threshold (QE not included).

## Data Availability

The original contributions presented in this study are included in the article. Further inquiries can be directed to the corresponding author.
